# Radiation exposure assessment of nuclear medicine staff administering [^177^Lu]Lu-DOTA-TATE with active and passive dosimetry

**DOI:** 10.1186/s40658-023-00592-1

**Published:** 2023-11-14

**Authors:** Mercedes Riveira-Martin, Lara Struelens, José Muñoz Iglesias, Werner Schoonjans, Olga Tabuenca, José Manuel Nogueiras, Francisco Javier Salvador Gómez, Antonio López Medina

**Affiliations:** 1 Genetic Oncology, Radiobiology and Radiointeraction Research Group, Galicia Sur Health Research Institute (IISGS), Vigo, Spain; 2 Department of Radiology, Rehabilitation and Physiotherapy, Medicine School, Complutense University of Madrid, Madrid, Spain; 3 Belgian Nuclear Research Centre (SCK CEN), Mol, Belgium; 4 Nuclear Medicine Department (SERGAS), Meixoeiro Hospital, University Hospital of Vigo, Vigo, Spain; 5 Nuclear Medicine Department (GALARIA), Meixoeiro Hospital, University Hospital of Vigo, Vigo, Spain; 6 Medical Physics and RP Department (GALARIA), Meixoeiro Hospital, University Hospital of Vigo, Vigo, Spain; 7 Department of Functional Biology and Health Sciences, University of Vigo, Vigo, Spain

**Keywords:** [^177^Lu]Lu-DOTA-TATE, Occupational exposure, Nuclear medicine, Equivalent dose

## Abstract

**Background:**

The use of lutetium-177 (^177^Lu)-based radiopharmaceuticals in peptide receptor nuclear therapy is increasing, but so is the number of nuclear medicine workers exposed to higher levels of radiation. In recent years, [^177^Lu]Lu-DOTA-TATE has begun to be widely used for the treatment of neuroendocrine tumours. However, there are few studies evaluating the occupational radiation exposure during its administration, and there are still some challenges that can result in higher doses to the staff, such as a lack of trained personnel or fully standardised procedures. In response, this study aims to provide a comprehensive analysis of occupational doses to the staff involved in the administration of [^177^Lu]Lu-DOTA-TATE.

**Results:**

A total of 32 administrations of [^177^Lu]Lu-DOTA-TATE (7.4 GBq/session) carried out by a physician and a nurse, were studied. In total, two physicians and four nurses were independently monitored with cumulative (passive) and/or real-time (active) dosemeters. Extremity, eye lens and whole-body doses were evaluated in terms of the dosimetric quantities Hp(0.07), Hp(3) and Hp(10), respectively. It was obtained that lead aprons reduced dose rates and whole-body doses by 71% and 69% for the physicians, respectively, and by 56% and 68% for the nurses. On average, normalised Hp(10) values of 0.65 ± 0.18 µSv/GBq were obtained with active dosimetry, which is generally consistent with passive dosemeters. For physicians, the median of the maximum normalised Hp(0.07) values was 41.5 µSv/GBq on the non-dominant hand and 45.2 µSv/GBq on the dominant hand. For nurses 15.4 µSv/GBq on the non-dominant and 13.9 µSv/GBq on the dominant hand. The ratio or correction factor between the maximum dose measured on the hand and the dose measured on the base of the middle/ring finger of the non-dominant hand resulted in a factor of 5/6 for the physicians and 3/4 for the nurses. Finally, maximum normalised Hp(3) doses resulted in 2.02 µSv/GBq for physicians and 1.76 µSv/GBq for nurses.

**Conclusions:**

If appropriate safety measures are taken, the administration of [^177^Lu]Lu-DOTA-TATE is a safe procedure for workers. However, regular monitoring is recommended to ensure that the annual dose limits are not exceeded.

**Supplementary Information:**

The online version contains supplementary material available at 10.1186/s40658-023-00592-1.

## Background

The number and variability of therapeutic and diagnostic procedures using radioactive isotopes has increased in recent years, as reflected in the growth of new radiopharmaceuticals recently approved or under development [1, 2]. However, this trend is also associated with an increase in the number of nuclear medicine (NM) workers exposed to radiation sources and a greater risk of radiation exposure. This is particularly relevant in the case of the extremities, as manual handling at short distances from sources can result in higher doses to the hands, especially in the case of beta-emitting sources [3, 4]. It is therefore necessary to assess occupational doses during these procedures to ensure that the introduction of novel radiopharmaceuticals into daily clinical practice does not have a negative impact on workers’ health.

Peptide receptor radionuclide therapy (PRRT) with the lutetium-177 (^177^Lu)-labelled tracer [^177^Lu]Lu-DOTA-TATE (^177^Lu-DOTATATE) was approved by the European Medicines Agency (EMA) [5] in 2017 and by the US Food and Drug Administration (FDA) in 2018 [6] after the success of the NETTER-1 clinical trial [7]. This novel radiopharmaceutical is currently available as a commercial preparation known as Lutathera® (AAA, a Novartis company) and is intended for the treatment of somatostatin receptor (SSTR) positive neuroendocrine tumours (NETs), including gastroenteropancreatic neoplasms (GEP-NENs). Several studies have shown that PRRT with ^177^Lu-DOTATATE (PRRT-Lu) is a highly effective treatment option for the control of metastatic, advanced, or unresectable progressive NETs, capable of improving the patients' health-related quality of life and slowing disease progression [8–11]. Its therapeutic use, together with its diagnostic partner [^68^ Ga]Ga-DOTA-TOC, has marked a watershed in the theragnostic landscape, enabling expression of the therapeutic target before therapy [12].

^177^Lu is one of the main isotopes of choice for theragnostic applications due to its decay characteristics, such as its dual beta (β) and gamma (γ) emissions, energy range and half-life. While the therapeutic effect is achieved through β-emission, the emitted photons provide information on the biodistribution of the radiopharmaceutical. However, this decay scheme also raises concerns about the radiation safety of those close to the patient, such as the nuclear medicine staff performing the treatment. Beta radiation can result in high doses to the hand (skin) when radioactive sources are handled closely [13], while gamma radiation can contribute to an increase in the dose received by the eye lens and/or effective dose to the whole body [14].

Recently, several efforts have been made to determine the external dose rates following the administration of ^177^Lu-DOTATATE in order to define acceptable patient release criteria based on the estimated radiation burden to relatives, visitors, caregivers and the underlying environmental issues [15–17], as well as the best clinical practices [18–21]. However, when it comes to personnel radiation burden, there are few studies published assessing the occupational exposure during the administration of this radiopharmaceutical [14, 22–24], and no studies were found considering doses to the eyes, extremities and whole-body combined.

The ORAMED project [25] was carried out between 2008 and 2011 and focussed in part on extremity and eye lens dosimetry of nuclear medicine staff. It was a major breakthrough in personal dose assessment, demonstrating that annual skin dose limits can be exceeded if radiation protection standards are low, and that extremity monitoring is essential in nuclear medicine. In addition, it was shown that the dosemeters used in the clinical practice for radiation protection, such as the regular ring or wrist dosemeters, may underestimate the maximum doses received to the hand, so a proper correction factor (CF) should be applied. However, the ORAMED project was mainly concerned with diagnostic radioisotopes and with ^90^Y as a therapeutic isotope, as ^177^Lu was only later approved. Following the ORAMED project, only a few studies have investigated occupational doses arising from ^177^Lu [26], only one of which focussed on the administration step of ^177^Lu-DOTATATE [13].

Recent studies have shown that the staff performing NM procedures are exposed to a wide range of radiation doses, depending on the patient, worker and activity [14, 17, 27], so a general quantification of radiation doses can be challenging. In addition, given the novelty of PRRT with ^177^Lu-DOTATATE, procedures are not fully standardised and can vary considerably from one institution to another. For example, whether the treatment is administered on an inpatient or outpatient basis [19], or whether a lead apron is required during administration. Therefore, there is still room for optimisation of occupational doses in this respect.

Additionally, the radiolabelled compound [^177^Lu]Lu-PSMA-617 (Pluvicto®, AAA) was approved by the FDA and EMA in 2022 for the treatment of metastatic castration-resistant prostate cancer. The final phase III clinical trial VISION showed that [^177^Lu]Lu-PSMA-617 reduced the overall mortality by 38% and disease progression by 60% in combination with the standard therapy [28]. Given these promising results, it will not be long before ^177^Lu-PSMA-617 is used in routine clinical practice, so prior assessment of ^177^Lu radiation exposure, as the one performed in this study, is necessary to ensure its safe incorporation. In addition, it has been reported that both ^177^Lu-DOTATATE and ^177^Lu-PSMA-617 have similar radiation exposure and blood clearance [23], so studies of the former may provide preliminary results for the latter. Furthermore, ^177^Lu is also being investigated to be used for the treatment of other somatostatin-receptor-expressing tumours beyond NETs, such as small-cell lung cancer and meningioma (NCT05142696, NCT03971461) [12], and even as an alternative for the treatment of thyroid cancer patients with low response to radioiodine (^131^I) [29].

Due to the paucity of studies and the emerging use of ^177^Lu in NM departments, this study aims to assess the radiation exposure to the hands, eye lens and whole body of nuclear medicine staff administering ^177^Lu-DOTATATE for the treatment of NETs using active and passive dosimetry. This study also intends to calculate the CFs required to estimate the maximum doses to the hand, and to identify the steps in the working procedure associated with higher dose rates.

## Materials and methods

### Radionuclide characteristics

The radiopharmaceutical [^177^Lu]Lu-DOTA-TATE, also known as ^177^Lu-Oxodotreotide or [^177^Lu]Lu-DOTA-(Tyr^3^)-octreotate is a ^177^Lu-labelled somatostatin analogue peptide conjugated with the bifunctional chelator DOTA and bound to the somatostatin affine peptide (Tyr^3^)-octreotate. The radionuclide ^177^Lu decays to hafnium-177 (^177^Hf) with a half-life of 6.7 days (162 h) emitting β^−^ particles with maximum energies of 497 keV (78.6%), 384 keV (9.1%) and 176 keV (12.2%) [30], resulting in a maximal (mean) soft-tissue penetration of the electrons of 1.7 mm (0.23 mm). It is therefore classified as a short-range β particle emitter [31]. The β decay is accompanied by low-to-medium energy γ-rays of 113 keV (6.6%) and 208 keV (11%) [30].

### Monitored staff and sessions

This study was conducted at the Meixoeiro Hospital (Spain) over a 22-month period. It encompassed a total of 32 sessions (administrations) and 10 patients who underwent NET treatment with Lutathera® (4 sessions, 8 weeks apart). All patients received the same fixed dose of 7400 MBq/cycle (i.e. 29.6 GBq/treatment) according to the predefined treatment with Lutathera®. However, according to the technical data sheet, the activity can be halved if the patient experiences adverse reactions in previous cycles, although this situation did not occur in any of the sessions included in this study. It should be noted that because some patients' sessions did not take place within the time period of the study, and others were administered by physicians/nurses not included, the administration of all four sessions (doses) was not monitored for all patients. In this case, the staff enrolled in the study were monitored while performing the administrations of 5 patients who received all 4 doses, 3 who received 3 doses, 1 who received 2 doses and 1 who received 1 dose (i.e. 32 sessions). At our hospital, the entire treatment is carried out by a NM physician and a nurse. On average, the administered activity per session was 7121 ± 105 MBq (range 6808–7289 MBq) and the residual activity was 50 ± 15 MBq (range 26–85 MBq) except for one session in which an accident caused the residual activity to be 148 MBq, which was removed from average data to be considered an outlier. A total of six right-handed workers were independently monitored in each session they participated in, both with cumulative (passive) and/or real-time (active) dosemeters: two physicians (P1, P2) and four nurses (N1, N2, N3, N4). A distinction is made between individual workers (P1, P2, N1, N2, N3 and N4) and groups of workers (physicians and nurses). Some of the sessions monitored with the active dosemeters were not monitored with passive dosemeters, so the number of sessions may differ between different types of detectors. The information on the number of sessions and staff is outlined in Table 1.

**Table 1:** Summary of the number of sessions recorded in this study with cumulative (passive) and real-time (active) dosimetry. Each set corresponds to the dosimetry equipment used in passive dosimetry

	Passive dosimetry			Active dosimetry		Apron
Staff	Set	Sessions (Protocol)*	Total activity (GBq)	Sessions (Protocol)*	Total activity (GBq)	
P1	–	–	–	2 (1)	14.7	No
	#1	4 (1)	28.9	12 (1)	85.7	Yes
	#2	4 (1)	28.3	7 (2)	49.8	
	#3	4 (1)	28.6			
	#4	7 (2)	49.8			
P2	#1	5 (1)	35.7	5 (1)	35.7	Yes
	#2	4 (2)	27.6	5 (2)	34.9	
N1	–	–	–	3 (1)	21.7	No
	#1	4 (1)	28.9	4 (1)	28.9	Yes
	#2	4 (2)	28.3	4 (2)	28.3	
N2	#1	2 (1)	14.1	2 (1)	14.1	Yes
N3	#1	9 (1)	64.3	9 (1)	64.3	Yes
	#2	6 (2)	42.3	7 (2)	49.6	
N4	–	–	–	2 (1)	14.2	Yes

*Protocols refer to the two different ways of administration using the gravity infusion method: Protocol 1 starts the infusion rate at 100 ml/h (30 min) and increases to 200 ml/h (15 min), while Protocol 2 starts at 100 ml/h (5 min) and increases to 400 ml/h (20 min)

The apron corresponds to a 0.5 mm lead equivalent apron

### Treatment protocol and shielding considerations

**Fig. 1:**
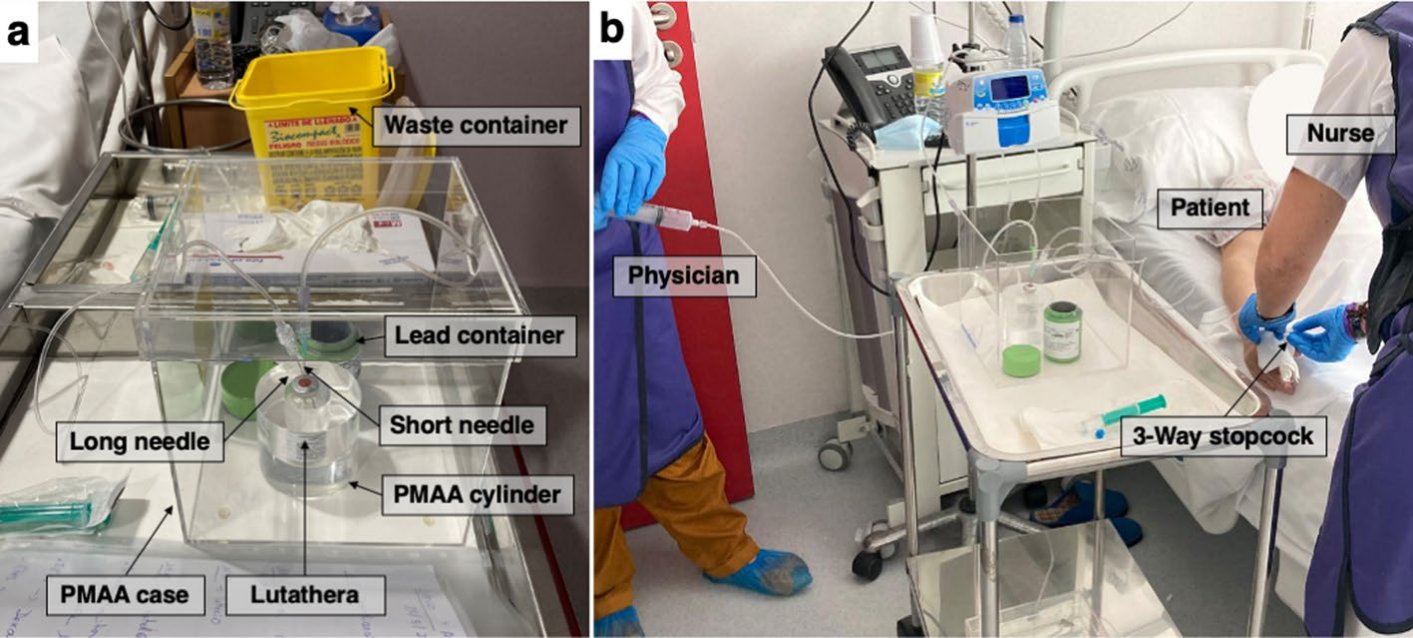
Set up during PRRT-Lu treatment: **a** configuration of the vial during administration, showing the Lutathera vial outside the original lead container and shielded with the PMMA cylinder and case, with the short and long needle inside it; **b** positions of physician, nurse and patient at the end of the administration, during air infusion

Lutathera® is a sterile ready-to-use solution for infusion with a volumetric activity of 370 MBq/ml at the reference date and time. It is delivered to the hospital on the day of each session as a single-dose vial of 20.5–25.0 ml, adjusted to yield an activity of 7400 MBq (200 mCi) per dose. The vial is enclosed inside a lead container (1.7 cm lead), which also contains a plastic foil covering the vial to attenuate the electrons. Prior to each administration, the patient is premedicated with antiemetics (Akynzeo 300 mg/0.5 mg, hard capsules). After 30 min, a commercially available amino acid solution is injected intravenously over 4 h. The treatment with ^177^Lu-DOTATATE entails the following steps:

1. *Activity verification* Prior to administration, the physician verifies the prescribed activity by removing the vial from the lead container and placing it into the radionuclide calibrator (CRC®-55tPET, Capintec, Inc.). This calibrator is equipped with a dipper to introduce the vial into the calibrator without handling. It undergoes a trimestral control to measure linearity, with a deviation of less than 5%, and the energetic response to high, medium, and low energies showing relative standard deviations and relative differences of less than 5%, as well as daily controls to check the stability of the chamber. To measure the activity of ^177^Lu, a calibration setting number, verified by measurements with a calibrated source, is automatically applied. No geometric considerations or adjustments are made to measure the activity of the Lutathera vial, as calibration is also performed using vials. The vial is handled with tweezers and behind a lead screen (50 mm lead equivalent screen with a built-in lead glass window of 21 mm lead equivalent). After checking the activity, the vial is returned to the lead container and the whole is placed inside a 3 mm thick polymethyl methacrylate (PMMA) box (Fig. 1a).
2. *Administration* Lutathera is infused intravenously (IV) following the gravity method (Additional file 1: Fig. S1), as recommended by the manufacturer [32]. In this method, two needles are inserted into the Lutathera vial, one short (0.8 × 40 mm, 21G × 1 1/2″) and one long (0.9 × 90 mm, 20G × 3.20″), with the long needle being the only one in contact with the radioactive liquid and the short needle connected to a sodium chloride solution (NaCl 0.9%). In this configuration, the vial is removed from the lead container and placed inside a 3 cm thick PMMA cylinder, which is held within the 3 mm thick PMMA box (Fig. 1a). Before the physician opens the lead container, the system is flushed with a 10 ml syringe to prevent overflow, which is important to minimise the time the staff is near the vial during infusion. During this step, the physician is responsible for handling the vial, inserting the needles and flushing the vials, while the nurse is responsible for selecting the infusion rate of the pump connected to the NaCl solution, taking the patient's IV and ensuring that there is no overflow of liquid. Once the flow is assured, both physician and nurse remain outside the administration room or behind a 30 mm lead equivalent movable screen placed in front of the patient's bed, to visually check the solution levels and prevent possible vial overflow or other potential issues. The most common position of the lead screen, as well as of the rest of items, is shown in the supplementary material both (Additional file 1: Fig. S2).
3. *Change in the infusion rate/other approaches* To ensure that the radiopharmaceutical is administered correctly and is tolerated by the patient, it is recommended to start the infusion at a slow rate for a few minutes and then increase the rate [32]. This change in infusion rate requires either the physician or the nurse (more often the nurse) to approach the patient, potentially increasing exposure. Two different protocols of the gravity method were used during the study: the first starts at 100 ml/h (30 min) and increases to 200 ml/h (15 min), while the second starts at 100 ml/h (5 min) and increases to 400 ml/h (20 min). The number of sessions performed with each protocol is shown in Table 1. During the session, other approaches within 1 m of the patient or the vial without a lead screen are also appreciated as dose peaks.
4. *End of administration* The infusion is stopped 15/20 min (first/second protocol) after the rate change. To extract as much of the radiopharmaceutical as possible, the pressure inside the vial is increased by the infusion of air at this stage. For this purpose, the physician replaces the saline line attached to the short needle with a 100 cm extension catheter with its end attached to a 10 ml syringe filled with air, through which it is slowly injected. The catheter allows the physician to keep the distance from the vial, reducing hand exposure. Meanwhile, the nurse is responsible for closing the three-way stopcock connected to the patient line, to prevent direct air entry (Fig. 1). This step also ensures that residual activity remaining in the catheter is minimal, thus reducing the risk of contamination due to bare-handed handling of the catheter. Once the stopcock is closed, the physician removes the needles from the vial, while the nurse stores the needles, catheters, and any potentially contaminated residue in a container, which is left to decay in a shielded room.
5. *Residual activity verification* The physician verifies the residual activity of the vial on the same radionuclide calibrator as in the first step. The vial is finally stored in the original lead container and inside a shielded cabinet to decay for six months (Additional file 1: Fig. S3).

In our NM department, Lutathera treatment is performed on an inpatient basis. The patient undergoes a gammagraphic image 24 h after the administration of ^177^Lu-DOTATATE, after which they can leave the hospital with the approval of the Radiation Protection (RP) department.

Throughout the treatment (from the initial administration to the residual activity verification) both the physician and the nurse wear a 0.5 mm lead equivalent apron to protect against gamma radiation. However, the first three sessions carried out at our institution were performed without the apron, allowing comparison of whole-body doses and dose rates with and without the apron (Table 1). Single-sided aprons present only one layer of 0.5 mm lead equivalent, while wrap around aprons with hook-and-loop closures present two layers of 0.25 mm lead equivalent, so that when closed, the total thickness is equivalent to an apron of 0.5 mm lead equivalent.

### Radiation monitoring equipment

**Fig. 2:**
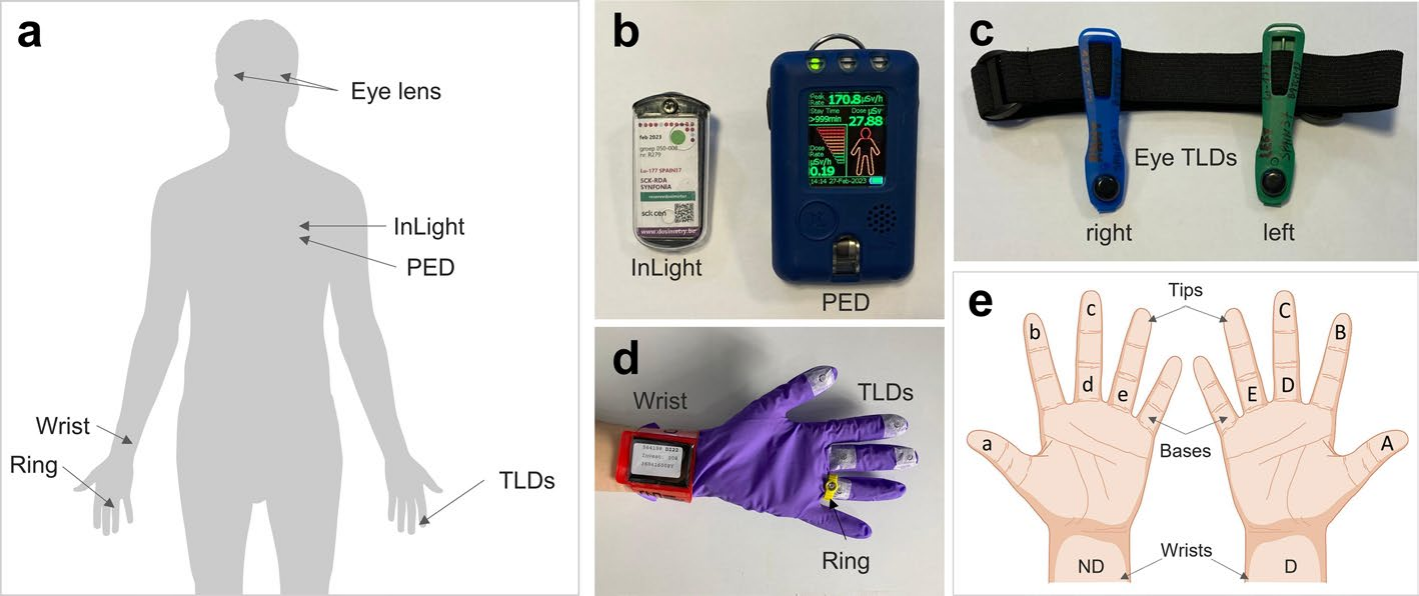
Radiation detectors used to monitor workers during ^177^Lu therapy used in this study: **a** schematic representation of the location of each dosemeter; **b** whole-body dosemeters, both passive (InLight or OSL) and active (PED); **c** eye lens dosemeters attached to the head band; **d** example of a hand with the TLDs attached to the gloves, the ring and wrist dosemeters, subsequently covered by regular nitrile gloves; **e** schematic representation of the location of the TLDs across the gloves

An Atomtex AT1123 plastic scintillator model (Atomtex®, Republic of Belarus) was used to record ambient dose equivalent rates (µSv/h) at different time points and positions within the administration room. It captures X-ray and gamma radiation in the energy range 15 keV–10 MeV and at dose rates between 0.05 and 10 Sv/h. In addition, personal electronic dosemeters (PEDs) (Tracerco™, London, United Kingdom) were used to record dose rates during each session received by both the physician and nurse (Fig. 2). They were placed at chest level and under the lead apron when worn. These detectors provide active dosimetry in terms of the dosimetric quantity Hp(10) accumulated in the X-ray and gamma radiation fields, integrated per minute, within the energy range 33 keV to 1.25 MeV and at dose rates between 0.1 μSv/h and 0.1 Sv/h.

Whole-body doses were also determined using passive dosemeters: an InLight badge (Landauer, Inc., Glenwood, IL) was placed at chest level and under the lead apron when worn (Fig. 2). These are Optically Stimulated Luminescence (OSL) dosemeters and provide both Hp(0.07) and Hp(10) quantities. They are sensitive to gamma and beta radiation in the energy range 16 keV–6 MeV and 0.7–2.3 MeV, respectively, and operate over a minimum detection limit of 50 µSv. The OSL badges were supplied and analysed by the Belgian Nuclear Research Centre (SCK CEN). This dosimetry system conforms to the IEC 62387 standard and has been approved by the Belgian Nuclear Control Authority (FANC).

To assess the dose distribution over the hands, five thermoluminescent detectors (TLDs) attached to nitrile gloves (200 μm-thick) were placed at different locations on both the dominant (D) and non-dominant (ND) hand, as shown in Fig. 2. These are LiF-based TLDs (LiF: Mg,Ti (MTS-N)) with the form of circular pellets with 4.5 mm diameter and 0.9 mm thickness [33]. They provide reliable measurements of doses in the range of μSv in terms of the dosimetric quantity Hp(0.07). These gloves are always covered with regular nitrile gloves to avoid cross-contamination. They are provided and analysed by the SCK CEN. The lowest detection limit (LDL) of these dosemeters (i.e. the lowest measurable dose) is defined as three times the standard deviation (SD) of the background detectors and ranged from 32 to 211 µSv for the hand dose measurements performed with ^177^Lu within this study.

Personal dose equivalent Hp(0.07) to the extremities was also measured with the regular ring and wrist dosemeters used in our institution for radiation risk assessment in NM, provided and analysed by the Spanish National Dosimetry Centre (CND) (Fig. 2). The ring dosemeter is the TLD DTX-RAD 707H-2 model (Thermo Fisher Scientific, Oakwood Village, USA), which is based on a 7 mg/cm^2^ thickness and 2 mm diameter detector of ^7^LiF:Mg,Cu,P [34]. This model allows for the measurement of Hp(0.07) by photons and beta particles in the range of 0.2 mSv–10 Sv. The ring dosemeter is placed at the base of the ring finger of the D or ND hand, over the TLD gloves and under the regular nitrile gloves with the detector facing the palm side. The wrist dosemeter is the DTX-100 model [34], which allows the measurement of Hp(0.07) as the average of four LiF:Mg,Ti detectors optimised for photons in the range 0.2 mSv–10 Sv. It is placed on the wrist of the D or ND hand (always the same as the ring) and directed towards the inner side of the hand. Doses below 0.1 mSv are not reported for either ring or wrist detectors.

Specific eye dosemeters (EYE-D, Radcard Poland) were used for monitoring eye doses. These are LiF:Mg,Cu,P TLD type (MCP-N) which allows Hp(3) measurements in the range of 10 μSv–10 Sv (Fig. 2). They were provided and analysed by SCK CEN. The LDL ranged from 15 to 93 µSv for the eye measurements performed with ^177^Lu within this study.

The SCK CEN dosimetry laboratory is accredited by the Belgian Accreditation Body (BELAC) for these TLD measurements in terms of Hp(3) and Hp(0.07) in the dose range from 50 μSv to 10 Sv according to ISO/IEC 17025 [35]. All the dosemeters used in this study were used in accordance with the indications defined in the IEC 62387 standard [36].

### Statistical analysis

Values are presented as mean ± SD or median [range]. The cumulative dose was normalised to the total activity (A), obtained as the sum of the administered activity measured in each session. Values were considered outliers if they exceeded 1.5 times the interquartile range (IQR). Boxplots are used to show the median, IQR, minimum and maximum values. Comparisons were made using hypothesis testing with the Mann–Whitney U test, which was chosen because some parameters did not follow a normal distribution, as assessed by the Shapiro–Wilk test. A 95% confidence level was assumed, so differences were considered statistically significant if the p value (*P value*) was less than 0.05. Statistical analysis was performed using RStudio software (v4.1.1) [37].

## Results

### Effect of lead apron

**Fig. 3:**
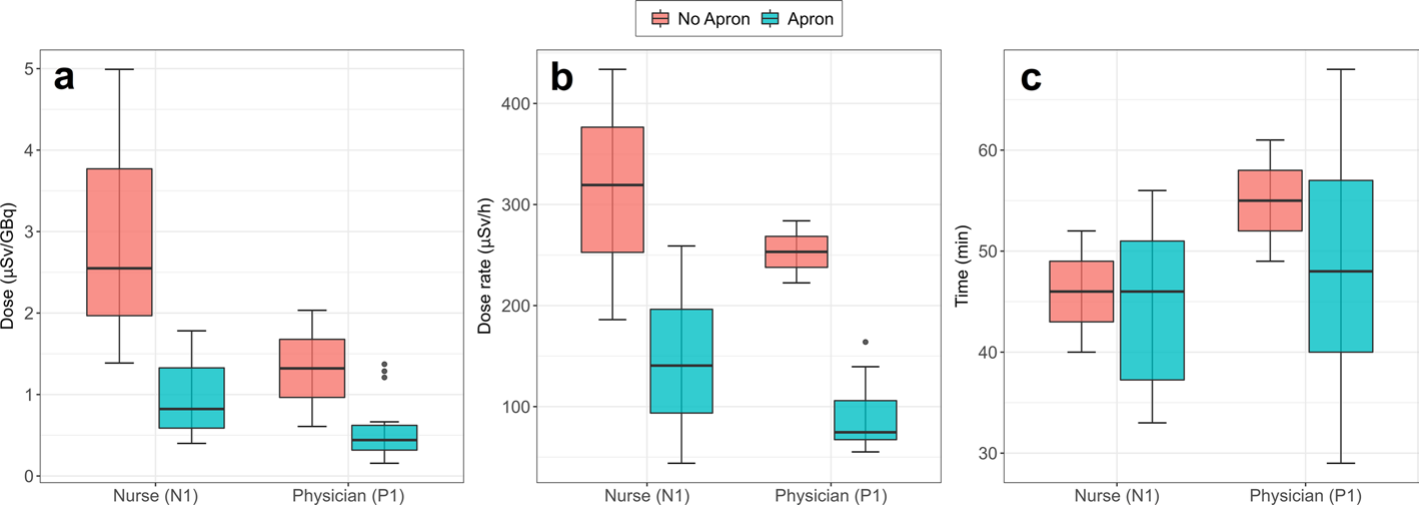
Boxplots comparing **a** whole-body dose, **b** maximum dose rates and **c** time of treatment between sessions with (blue) and without (red) lead apron for nurse (N1) and physician (P1). *N* = 29 sessions were monitored with apron for both physician and nurse, whereas *N* = 2 and *N* = 3 sessions for the physician and nurse, respectively, without apron. The middle line represents the median value, the top and bottom bars the maximum and minimum values, respectively, and the box limits the IQR. Single points are outliers

The effect of the lead apron was studied for two workers (P1 and N1) by comparing the dose rates and the cumulative doses measured with PEDs during the first sessions conducted at our institution without apron (*N* = 2 and *N* = 3 by the physician and nurse, respectively) and the remaining sessions performed by the same workers wearing an apron (*N* = 29 each) (Table 1). It was obtained that lead aprons reduced the median maximum dose rates and cumulative doses by 71% and 69% for the physician, respectively, and by 56% and 68% for the nurse. On the contrary, it was observed that sessions with an apron did not take more time. Results are shown in Fig. 3. Numerical results are summarised in the additional file (Additional file 1: Table S1).

### Dose rates

Ambient dose rates were measured at three different time points: after the initial administration (Admin.), at the change of the infusion rate (IR), and at the end of the administration (End). These were also measured at three different positions: at the surface of the PMMA box (Vial), 0.5 m away from the patient's chest with no lead screen in between (Patient) and behind the lead screen (Screen). These values are summarised in Table 2 for 5 sessions with the first protocol and 7 sessions with the second. Dose rates decreased from administration to the end of the treatment in the vial and followed the opposite trend at 0.5 m from the patient. However, dose rates behind the lead screen remained constant, with median and mean values of 0.39 µSv/h and 0.49 ± 0.29 µSv/h, respectively. Background radiation was not removed from these measurements and was found to be 0.2 µSv/h. Dose rates at the beginning (0 min) and at the end of administration are similar for both protocols, but change substantially in the change of the infusion rate, as in the second protocol it is performed in the first 5 min. The relative change from administration up to the end of treatment follows a similar reduction about 98% for both protocols.

**Table 2:** Ambient dose rates recorded with the Atomtex near the vial, at 0.5 m of the chest of the patient and behind the lead screen at three different time points: administration (Admin.), the change in the infusion rate (IR) and the end of administration (End)

Protocol	Step	Initial activity (MBq)	Time stamp (min)	Dose rate (µSv/h)		
				Vial	Patient	Screen
1 (5 sessions)	Admin	7130 ± 99	0	2320 [1930–3100]	37 [23–124]	0.5 [0.3–0.9]
	IR		30	138 [103–235]	410 [30–510]	0.4 [0.4–0.5]
	End		45	20 [9–31]	600 [50–680]	0.4 [0.3–0.5]
2 (7 sessions)	Admin	7120 ± 63	0	1960 [1690–2200]	48 [20–86]	0.4 [0.3–1.1]
	IR		5	1300 [370–1400]	184 [50–940]	0.4 [0.3–1.0]
	End		30	35 [16–102]	580 [430–670]	0.3 [0.3–0.4]

The results are divided by protocol

For each worker, the residual activity (measured by the physician), duration of treatment, the normalised cumulative dose and peak dose rates obtained with PEDs, were compared between protocols 1 and 2. This comparison was not possible for N2 and N4 as they only attended sessions using one protocol. Mann–Whitney U test showed that the residual activities measured by P1 were significantly different (*P value* < 0.01), with an average of 49.4 ± 7.1 MBq (protocol 1) and 34.0 ± 6.8 MBq (protocol 2), but non-significant for P2 (*P value* = 0.28), averaged 68.5 ± 21.6 MBq (protocol 1) and 56.5 ± 7.5 MBq (protocol 2). The peak dose rates, as well as the normalised cumulative dose showed non-significant differences between both protocols (*P value* > 0.05) for any of the workers. However, the treatment time showed a significant decrease from the first to the second protocol for all workers. Mean values and *P values* for each variable and protocol are shown in the additional file (Additional file 1: Table S2).

From now on, the analysis will be performed without distinguishing between protocols, as no significant differences in doses and dose rates recorded with PEDs were found. For each session, the highest dose rate value was selected as the peak dose rate for each worker, and these values were then averaged over all sessions performed by the same person. Table 3 shows the median, minimum and maximum of this peak dose rate for each worker. On average, physicians and nurses reached peak dose rates of 93 ± 31 µSv/h and 113 ± 56 µSv/h, respectively, and received normalised whole-body doses of 0.60 ± 0.06 µSv/GBq and 0.64 ± 0.20 µSv/GBq, respectively.

**Table 3:** Peak dose rates and normalised cumulative doses recorded with PEDs for each worker and averaged over both groups. Values are shown as median [range] and mean ± SD

Worker	Sessions	Dose rate (µSv/h)	Hp(10)/A (µSv/GBq)
P1	19	74.6 [55.1–163.9]	0.56
P2	10	97.3 [55.4–170.2]	0.65
N1	8	140.6 [43.9–259]	0.97
N2	2	103.0 [67.6–138.3]	0.66
N3	16	107.9 [31.1–205.8]	0.51
N4	2	63.2 [61.3 – 65.2]	0.47
Physicians	29	93 ± 31	0.60 ± 0.06
Nurses	28	113 ± 56	0.64 ± 0.20

**Fig. 4:**
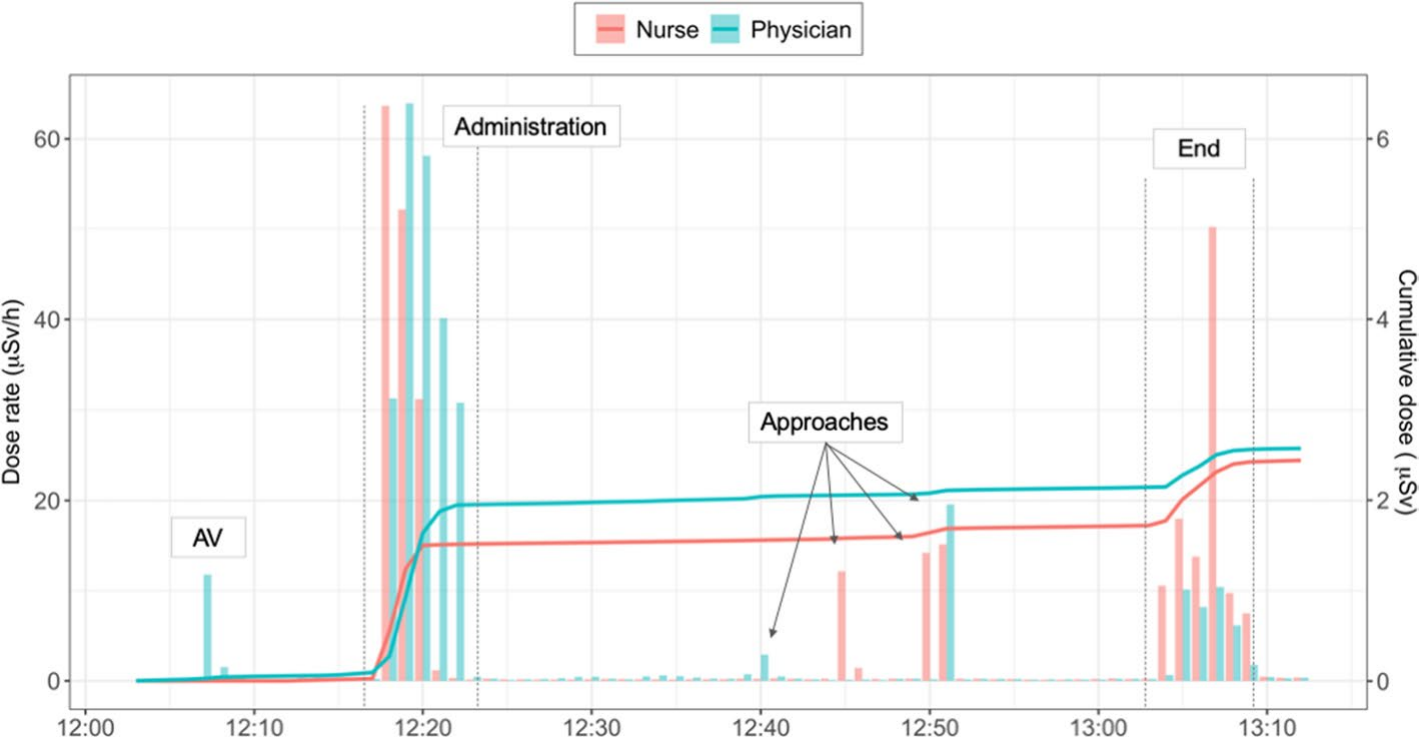
Example of dose rates and cumulative doses recorded with PEDs during one session of PRRT-Lu performed by one physician and one nurse. Dose rates are represented as vertical bars and cumulative dose as lines. Dose rates are measured in µSv/h integrated per minute. The different steps are shown (AV: activity verification, End: end of administration)

Since PED detectors allow the dose received to be determined in real-time, the relative time and dose burden of each step of the treatment was studied. As explained in the previous section, the following steps were considered: the activity verification (“AV”) and the post-treatment residual activity verification (“post-AV”), the initial administration (“Admin.”), the end of treatment (“End”), several approaches to less than one metre to the vial or patient with no lead screen in between, including the approach to change the infusion rate (“Approach”), and the time spent outside the patient's room and/or behind the lead screen (“Out”). Figure 4 illustrates an example of the dose rates and cumulative doses of both physician and nurse recorded during one complete treatment session, which shows that the administration and the end of administration entail the highest dose rates and that several approaches to less than 1 m of the patient or vial are also reflected as dose peaks.

**Fig. 5:**
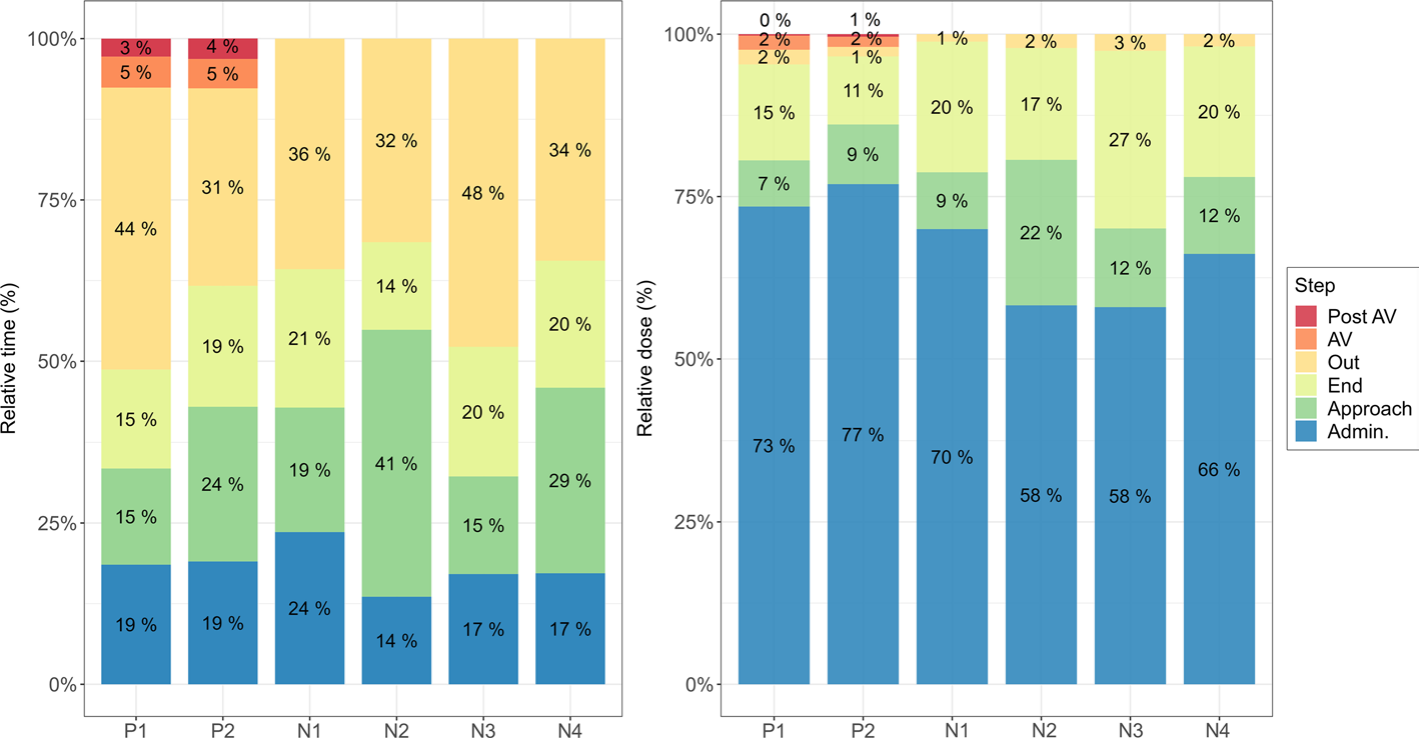
Relative time (left) and dose (right) associated to each step for each physician (P1, P2) and nurse (N1, N2, N3, N4), indicated by different colours

The relative time and whole-body dose measured with PEDs in terms of Hp(10), averaged over all the sessions carried out by each worker, are shown in Fig. 5. These values are also averaged for both groups of workers and for all workers (Table 4). The initial administration involves the highest dose burden (69%); whereas the time spent on this step is not the highest (19%), reflecting the high dose rates received. After the first administration, the end of the treatment also accounts for a significant proportion of the total dose for both workers (19%). On the contrary, both spend most of their time outside the patient's room or behind the lead screen (41%), but the dose associated with this is among the lowest (2%). It was also found that approaches within one metre of the vial or patient (without the screen), which includes approaching the patient to change the infusion rate, accounted for 10% of the total dose and 18% of the time. Of all the steps, both initial and post-treatment activity verifications resulted in the lowest doses for physicians.

**Table 4:** Relative percentage of time and dose associated with each step for the group of physicians, nurses and averaged over both

Step	Physician		Nurse		All workers	
	Time (%)	Dose (%)	Time (%)	Dose (%)	Time (%)	Dose (%)
Admin	19 ± 8	75 ± 13	19 ± 8	63 ± 14	19 ± 8	69 ± 15
Approach	18 ± 13	8 ± 11	19 ± 13	12 ± 15	18 ± 13	10 ± 13
End	16 ± 6	13 ± 8	20 ± 6	24 ± 10	18 ± 6	19 ± 10
Out	39 ± 16	2 ± 1	42 ± 12	2 ± 1	41 ± 14	2 ± 1
AV	5 ± 1	2 ± 5	−	−	5 ± 1	2 ± 5
Post AV	3 ± 2	0.3 ± 0.6	−	−	3 ± 2	0.3 ± 0.6

### Whole-body doses

Whole-body doses with OSL (passive) dosemeters showed Hp(0.07) and Hp(10) values below the LDL (50 µSv) for almost all workers except for P1 (#2 and #4) and N2 (#1) (Table 5), with a very high Hp(0.07) value for P1 #4. The average Hp(10) of both workers is 11.6 ± 2.9 µSv/GBq. On the other hand, Hp(10) doses recorded with PEDs showed values below/on the order of 1 µSv/GBq for all workers. According to PEDs, the average Hp(10) for physicians is 0.60 ± 0.07 µSv/GBq and for nurses 0.72 ± 0.26 µSv/GBq, being not significantly different (*P value* = 0.42), with an average value of 0.65 ± 0.18 µSv/GBq.

**Table 5:** Whole-body (Hp(10) and Hp(0.07)), extremities (Hp(0.07)) and eye lens (Hp(3)) doses normalised to the total handled activity (µSv/GBq) obtained for each worker and set of detectors

Staff	Set	Normalised dose (µSv/GBq)*									
		Hp(10)/A, Hp(0.07)/A Whole-Body			Hp(0.07)/A Extremities					Hp(3)/A Eye lens	
		Active	Passive		TLD (Gloves)†		CND			Left	Right
		Hp(10)	Hp(10)	Hp(0.07)	ND	D	Ring	Wrist	Hand		
P1	#1	0.61	–	–	41(a)	19(B)	–	6.9	D	2.3	2.1
	#2	0.63	13.7	13.6	66(b)	22(C)	17.7	10.6	D	27.1	23.2
	#3	0.48	–	–	47(b)	20(B)	14.0	7.0	D	–	0.6
	#4	0.53	–	131.7	53(b)	25(A)	14.0	4.0	ND	1.4	1.5
P2	#1	0.65	–	–	34(c)	70(A)	8.4	8.4	D	1.6	2.0
	#2	0.64	–	–	901(a)	1145(B)	108.8	39.9	ND	2.8	–
N1	#1	0.83	–	–	–	–	–	–	D	1.2	2.5
	#2	1.11	–	–	33(b)	14(B)	10.6	3.5	ND	–	–
N2	#1	0.66	10	11	164(e)	15(A)	–	14.2	D	–	–
N3	#1	0.60	–	–	8(a)	11(E)	7.8	4.7	D	–	1.0
	#2	0.41	–	–	8(a)	11(B)	61.4	2.4	ND	–	–

*Empty values (−) represent results below the lowest detection limit (LDL). For OSLs this value is 50 µSv and for ring and wrist dosemeters is 0.1 mSv

†Shown the maximum dose across the hands and the position in which it is received (Fig. 2e)

### Extremity doses

**Fig. 6:**
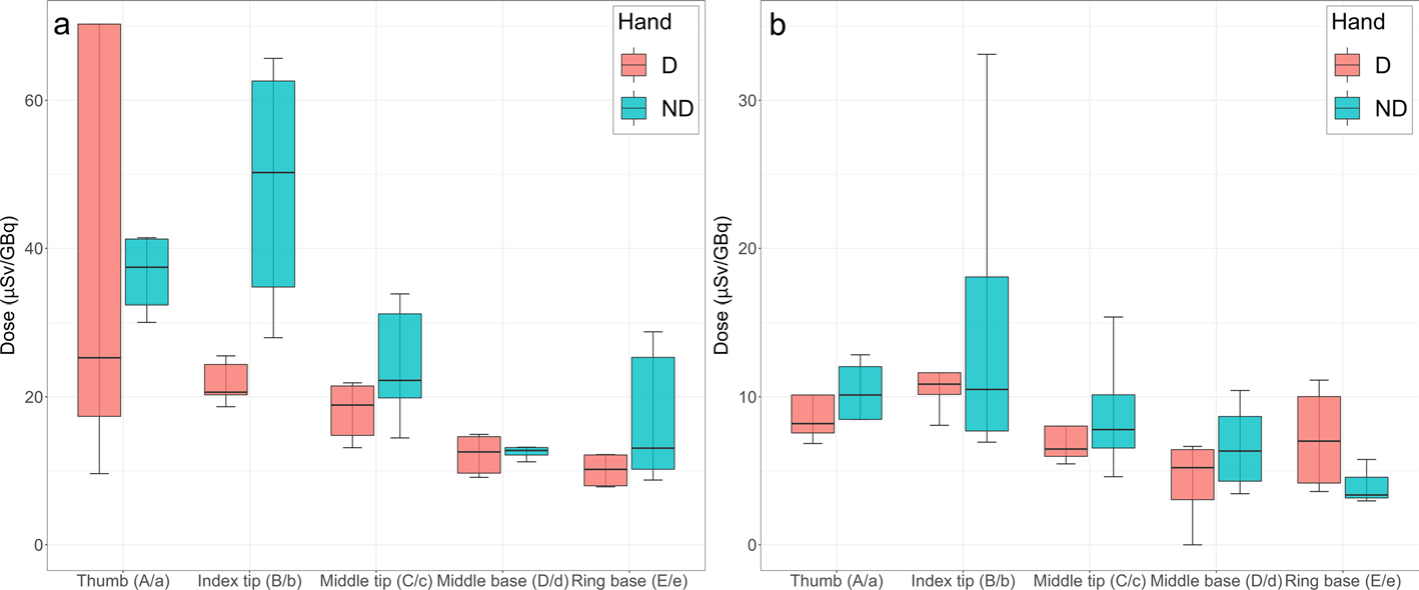
Boxplot of hand doses by location and hand for** a** physician and** b** nurse. Outliers were excluded for visualisation purposes

Table 5 shows the most exposed position (positions detailed in Fig. 2e) and its corresponding normalised dose value for each worker and set, both for the dominant (D) and non-dominant (ND) hand. Hp(0.07)/A values measured with the CND's ring and wrist dosemeters are also shown, together with the hand in which they were worn. Figure 6 shows the normalised dose values by hand and location for both groups of workers. It was obtained that the thumb (a/A) and the tip of the index finger (b/B) are the locations receiving the highest doses most frequently, in both hands and for both groups of workers. In addition, doses received on the ND hand appear to be higher than on the D hand, this difference being more acute for physicians than nurses. However, according to the Mann–Whitney U test performed between doses in the D and ND hand for each location, these differences are not statistically significant for either physicians or nurses (*P value* > 0.05) (Additional file 1: Table S3). Overall, the doses received by physicians are significantly higher than those received by nurses in all locations (*P value* < 0.05), except at the bases of the middle (*P value* = 0.09) and ring (*P value* = 0.48) finger of the D hand (Additional file 1: Table S4).

The maximum doses (median and range) by hand and by group of workers are summarised in Table 6. To obtain these values, the average of the normalised dose for each location is calculated for each worker over the different sets that he/she used. For each hand, the maximum value of the 5 locations was taken as his/her maximum dose. Then, the median of these maximum doses, by hand, is calculated for both groups of workers. The same was done for the ring and wrist dosemeters. Outliers were excluded from the calculation of this median, which were position e from N2 set #1 and all positions from P2 set #2, as well as the ring and wrist values, according to the outlier criterion. A more detailed explanation on the calculation of these values is presented in (Additional file 1: Table S5).

**Table 6:** Median and range of maximum normalised doses received to the hands for Physicians and Nurses for D, ND hand. Also for the CND ring and wrist dosemeters. Outliers have been excluded

Staff	Maximum normalised Hp(0.07) (µSv/GBq)			
	TLD (Gloves)		CND	
	D	ND	Ring	Wrist
Physicians	45.2 [20–70.3]	41.5 [33.8–49.2]	11.8 [8.4–15.2]	7.8 [7.1–8.4]
Nurses	13.9 [9.5–14.8]	15.4 [8.5–33.1]	9.2 [7.8–10.6]	3.5 [3.5–3.5]

Finally, a correction factor (CF) was calculated for each dosemeter set as the ratio of the maximum dose to the dose received at the positions suitable for routine dose measurements (i.e. the base of the fingers). Also, at the positions of maximum exposure (tips of the index finger and thumb), as performed by Carnicer et al. [38]. For this purpose, the CF was individually calculated for each dosemeter set and then the median and range were obtained by hand of each group of workers. Results are shown in Table 7.

**Table 7:** CFs for each group of workers, obtained as the quotient of the maximum dose and the dose at other positions

Staff Hand	CF (maximum dose/dose at other positions)					
	Thumb (a/A)	Index tip (b/B)	Base middle (d/D)	Base ring (e/E)	Ring (CND)	Wrist (CND)
*Physician*						
D	2.9 [1.0–4.3]	2.6 [2.2–3.2]	4.9 [1.4–5.8]	5.9 [5.4–6.4]	3.7 [3.4–8.4]	6.5 [6.0–8.4]
ND	1.4 [1.0–2.2]	1.0 [1.0–2.5]	4.8 [3.2–5.3]	4.8 [1.4–6.3]	3.8 [−]	13.3 [−]
*Nurse*						
D	1.4 [1.0–4.8]	1.4 [1.0–2.4]	2.7 [1.7–5.2]	2.3 [1.0–7.6]	1.4 [−]	1.7 [1.1–2.4]
ND	1.3 [1.2–2.8]	1.4 [1.2–1.6]	2.8 [1.5–4.1]	3.7 [3.2–5.7]	2.1 [1.0–3.1]	7.0 [4.6–9.4]

### Eye lens

Individual Hp(3)/A values are shown in Table 5. The median estimations show that physicians received 1.94 [1.36–2.83] µSv/GBq in the left eye and 1.76 [0.63–2.15] µSv/GBq in the right eye, this difference being not statistically significant (*P value* = 0.48). For nurses, two dosemeters showed values larger than the LDL in the right eye, resulting in a median of 1.76 [1.0–2.53] µSv/GBq. However, in the left eye only one value is available (1.18 µSv/GBq), so statistical comparison would be inaccurate.

The maximum doses (median and range) by group of workers are 2.02 [1.84–2.20] µSv/GBq and 1.76 [1.00–2.53] µSv/GBq for physicians and nurses, respectively. For this calculation, first the mean value of each eye over the different dosemeter sets per worker was obtained, and then the maximum value between right or left was selected. The median was then calculated from these maxima for each group of workers. Both eyes from P1 set #2 are considered outliers and excluded from the median calculation. However, this value is not neglected, as it represents potential harm.

### Estimation of the annual sessions limit

On the basis of the maximum normalised dose values obtained for Hp(10), Hp(0.07) and Hp(3) and the ICRP limits established by Council Directive 2013/59/Euratom (20 mSv, 500 mSv and 20 mSv respectively), the maximum number of sessions (cycles) of 7.4 GBq that could be performed in one year was calculated for each worker. These calculations are based on the measurements of workers wearing the 0.5 mm lead equivalent apron. Outliers were included in these estimates. In terms of Hp(10) measured with PEDs, the data show that there are no restrictions needed on the number of sessions per worker, as they range from 2000 to 4000 year. According to the two measurable doses recorded with OSL, the number of sessions would be limited to 197 by the P1 physician's maximum whole-body dose and to 282 for N2 doses. Extremities and eye lens session limit estimates are shown in Table 8. The estimated session limit based on Hp(0.07) exhibits a wider range, with 59 being the most restrictive number of sessions for P2. Finally, based on Hp(3) measurements, the most restrictive limit would be estimated in 100 sessions for P1, although the other workers present higher values.

**Table 8:** Maximum normalised dose values for each patient of extremities, eye lens, and the annual session limit estimated based on these values and the annual dose constraints

Staff	Extremities (gloves)		Eyes	
	Max. Hp(0.07)/A (µSv/GBq)	Session limit	Max. Hp(3)/A (µSv/GBq)	Session limit
P1	66.0	1024	27.1	100
P2	1145.0	59	2.8	955
N1	33.0	2048	2.5	1068
N2	164.0	412	–	–
N3	11.0	6143	1.0	2713

## Discussion

The clear patient benefits of PRRT with ^177^Lu-DOTATATE have inevitably increased the number of interventions and studies on this isotope and its theragnostic couples, such as ^68^ Ga-DOTATOC [12, 26, 39]. However, there are still some challenges regarding its use, such as lack of trained personnel or fully standardised procedures [12], which may result in high doses to the NM staff. In consequence, this study aims to analyse the occupational exposure during PRRT-Lu, using both passive and active dosimetry.

The use of lead aprons in NM departments is not as clear-cut as in interventional radiology, existing both supporting and opposing arguments for their use [40], such as the time extension that their heavy weight may cause, their diminishing attenuating ability with increasing photon energies, or the potential production of Bremsstrahlung radiation [41]. Nevertheless, the latter is less important in the case of ^177^Lu than other therapeutic pure beta emitters, such as yttrium-90 (^90^Y), due to the lower energy of its beta spectrum [22]. On top of that, the shielding material with the lowest atomic number (PMMA) is the first in contact with the nuclide, so that the Bremsstrahlung production is minimised and only the remaining gamma radiation from the decay is stopped by the apron. Moreover, these photons (113 and 208 keV) are less energetic than those emitted by other radioisotopes used in positron emission tomography (PET), such as ^18^F (511 keV) or ^68^ Ga (1077 keV), for which the use of the apron is not recommended due to the low attenuation capacity at these energies. In our hospital, the use of lead apron was introduced after the first few sessions. Therefore, with only 3 sessions performed without apron, a thorough hypothesis testing was not possible. Still, it was found that the doses and dose rates received with the apron were substantially lower than without, while the treatment time remained the same. Sghedoni et al. [22] compared the physician's personal equivalent doses over and under the lead apron during labelling and administration of ^177^Lu-DOTATOC and ^90^Y-DOTATOC. They obtained that radiation was almost completely stopped by the apron for ^90^Y, as it is a pure beta emitter and PMMA was used first, whereas the ^177^Lu-gamma emission was just partially attenuated, so they suggested that its use during ^177^Lu procedures should not be considered essential. However, they used a 0.25 mm lead equivalent apron, while those used in our study were 0.5 mm, so their gamma-stopping efficacy is higher. Manogue et al. also recommend the use of a lead apron during ^177^Lu-PSMA-617 therapy [42]. Accordingly, this study suggests that the use of a 0.5 mm lead equivalent apron during ^177^Lu-DOTATATE administration is recommended, as we experienced dose rates and whole-body doses reduction without prolongation of time.

It has been obtained that the dose rate behind the lead shielding remains at a constant value about 0.4 µSv/h. However, it should be noted that the natural background radiation plays an important role in this measurement. A dose rate value of 0.2 µSv/h was measured before the beginning of each session, which is consistent with the natural gamma dose rate value in the south of Galicia (Spain) [43]. Therefore, staying behind the lead screen can be associated with a dose rate of 0.2 µSv/h. In addition, the fact that dose rates decrease with time near the vial and increase near the patient is expected, as they follow the flux of the radiopharmaceutical. Dose rates measured immediately after the infusion of ^177^Lu-DOTATATE at 1 m from the chest of the patient reported in the literature were found to range (on average) from 16.2 to 34.9 µSv/h [14, 19, 44], and 48 µSv/h for 7.4 GBq of ^177^Lu-PSMA-617 [27]. Our results are in line with these studies, although within a larger range. This can be explained by the variability that certain patient-related factors may introduce, such as the body mass index (BMI) as described by Bellamy et al. [17], and by possible variations in timing and distance at the moment of measurement.

Ambient dose rates were similar for both protocols except at the change of the infusion rate, which is expectable as the timing is different. Protocol 2 takes less time than protocol 1, so although the dose rates are higher in the 5 min approach, total time is shorter, which can explain why dose rates and normalised doses received by each worker from both protocols showed no significant differences. Nevertheless, the second protocol has been adopted in our hospital from now on, as we obtained no significant differences in radiological protection, it is faster and is the actual protocol recommended by the EMA [45].

The administration step involves the highest dose rates and accounts for the majority of the cumulative dose, followed by the end of treatment, so there is room for optimisation in these steps if further dose reduction is required. Also, approaches to the patient or vial beyond the lead screen should be reduced as much as possible, as doses were not found to be negligible. Although the dose rates for both groups of workers are approximately the same, the nurses exhibit slightly higher values than the physicians, except for N4, who also presents lower values than the other nurses. Firstly, it should be noted that the administration of therapeutic lutetium is a longer and more complex process than the administration of a diagnostic radiopharmaceutical, as accidents are more likely to occur and therefore different sessions can be very different from each other. The value of the cumulative dose and dose rates also depends on the behaviour of the individual worker, with some considering it a priority to be closer to the patient or vial to ensure that the liquid does not spill, and others considering it more important to perform the procedure quickly and keep as much distance as possible, which can also vary between different sessions, even if they are carried out by the same person. In addition, patient behaviour can also introduce a lot of variability, since if the patient experiences discomfort, they will call the staff more often (so there will be more approaches) and exposure will increase. Therefore, it is most likely that the difference in dose rates between N4 and the other nurses is due to variability between sessions, as well as in the number of sessions performed and their behaviour during them. There is no significant difference in the level of training between nurses and they have approximately the same years of experience. On the other hand, physicians are responsible for vial and needle manipulation during administration and at the end of administration, so they receive higher doses on their hands compared to nurses due to the contribution of short-range beta emission. However, as nurses are in charge of patient care and monitoring, they make more approaches to the vial/patient (as can also be seen in Fig. 5) and thus spend more time close to radioactive sources. It is therefore to be expected that doses and dose rates obtained with the PED, which relate to whole-body doses and do not take account of betas, will be higher for this group of workers.

For most measurements there is good agreement between Hp(10)/A measured with PED and OSL dosemeters, as in these cases the dose measured with PED are below the LDL of 50µSv of OSLs, as well as OSLs. Only two sets showed higher Hp(10)/A values with OSL (sets P1#2 and N2#2). However, these sets were used during the same period of the measurement campaign, and they exhibited very high background doses due to long time between reset and readout and potentially due to transit doses. Besides, the correction with a single background dosemeter is imprecise, so there is a significant chance that the dose is due to fluctuations in background doses. Finally, it should be noted the high value of Hp(0.07) obtained in set P1#4. The fact that Hp(0.07) is so high, but that no value was obtained for Hp(10), indicates that this is a dose from a beta field, which is not measured by PEDs, so it is likely to have been slightly contaminated. It was verified that the physician’s monthly personal dosemeters, used during the same period as the measurement campaign and also placed at chest level, did not show high doses of Hp(10), which reinforces the option of contamination. On the other hand, the results obtained with PEDs are consistent with other studies that have measured Hp(10), also with electronic dosemeters. In the study by Sghedoni et al. [22] the physician’s Hp(10) was obtained during administration of ^177^Lu-DOTATATE in similar conditions to the present study (i.e. with electronic dosemeters, under apron). They resulted in 6.6 [1–24] µSv, which normalised to the injected activity (an average of 5.5 GBq to 3–4 patients) is 0.40 [0.06–1.45] µSv/GBq, in line with our results with PEDs. Other studies also show similar values (0.36 ± 0.16 µSv/GBq [23] and 0.24 ± 0.05 µSv/GBq [14]), although no information is given on the use of aprons. A study involving administration of ^177^Lu-PSMA-617 obtained 0.60 ± 0.05 µSv/GBq for physicians behind a lead shield [46]. Additionally, in a previous study on Hp(10) doses during manipulation of ^68^ Ga-DOTATOC [47] it was concluded that electronic dosemeters could underestimate the dose due to the non-linear response of the Geiger-Müller detector at the ^68^ Ga photon energies (1077 keV) and the pure beta field. However, the linear response of PED is straighter in the range of the ^177^Lu-photon energies (208 keV) [48]. In addition, the fact that both detectors are placed under the apron also introduces uncertainty, since as demonstrated in previous studies [49] the response of passive dosemeters is more influenced than that of active dosemeters in the Hp(10) reading, so it would be interesting to perform double dosimetry (detectors above and below the apron). Since no literature was found on whole-body doses from ^177^Lu administration with OSL dosimetry, further measurements would be needed. These results also highlight the need for caution when comparing doses from different dosimetry systems, especially when the LDLs are different or unknown.

Extremity dosimetry is a concern in NM practices, but according to a recent review by Kollaard et al. [26], only a few publications have addressed finger exposure with novel radiopharmaceuticals, with only three articles on ^177^Lu found at the time [13, 22, 24]. These studies presented Hp(0.07)/A values ranging from 10 to 66 µSv/GBq, which are similar to those presented in our study for both physicians and nurses excluding outliers (8–70 µSv/GBq), being 45.2 [20–70.3] µSv/GBq the highest maximum normalised dose obtained for physicians and 15.4 [8.5–33.1] µSv/GBq for nurses. As seen from Fig. 6, doses in the ND hand are qualitatively higher than in the D hand for both physicians and nurses. However, according to Table 6, physicians presented higher maximum normalised doses in the D than in the ND hand. This can be explained because the calculations in Table 6 are very conservative, as they have been obtained from the mean maximum values of each worker, therefore providing an estimate of the worst-case scenario. In this case, the values of set #2 of P2 have been considered outliers (Additional file 1:Table S5), and the thumb position of the D hand (A) of set #1 for the same physician, although not counted as an outlier, also shows a very high value compared to the rest (70 µSv/GBq versus a range of 19–25 µSv/GBq for P1, as seen in Table 5). Therefore, this value counts as the highest for this worker, and carries much weight when calculating the median with the P1 value, which shows a mean maximum value in the D hand of 20.1 µSv/GBq, giving a median of 45.2 µSv/GBq in the D hand (Table 6 and/or Additional file 1: Table S5), higher than the ND hand. However, according to the individual values for each position (Additional file 1: Table S5), without considering the maximum doses for each worker, the ND hand shows median values ranging from 11 to 47 µSv/GBq and the D hand from 10 to 21.3 µSv/GBq for physicians, and 3–11 µSv/GBq and 6–11 µSv/GBq for the ND and D hand, respectively, for nurses. Therefore, it is observed that the dose deposition is higher in the ND hand than on the D. In addition, this difference is more evident for physicians as they are responsible for manipulating the vial, which is generally done with the ND hand, so that they can handle the rest of the equipment (needles, plunger of the syringe, etc.) with the D hand. These results match with the fact that physicians received statistically significant higher doses than nurses in almost all locations (Additional file 1: Table S4). Nevertheless, although doses are qualitatively higher on the ND hand, especially for physicians, no statistically significant differences were found between both hands for either physicians or nurses. In addition, no significant differences were found between doses on the D and ND for any location (Additional file 1: Table S3), although the tip of the thumb and index finger show higher values in both hands for both physicians and nurses. These results are in contrast to those obtained after the manipulation of other radiopharmaceuticals, such as ^68^ Ga [47], ^18^F or ^99m^Tc, as shown in the ORAMED study [25, 38], in which the ND is significantly more exposed than the D hand. This is explained due to the direct handling of the vial/syringe and the pure beta emission of these isotopes. With the gravity method used to infuse ^177^Lu-DOTATATE, the vial is always inside the PMMA shield, so the physician does not need to hold the vial directly except in rare occasions, which explains why there is less difference between the D and ND hands than with other radiopharmaceuticals. Also, because of the dual emission of ^177^Lu, in contrast to the pure beta emission of ^68^ Ga or ^18^F, the dominant hand is also exposed to the gamma radiation. In addition, the administration of these diagnostic radiopharmaceuticals is faster and entails few steps, taking no more than 2–3 min of vial or syringe handling. This is opposed to the administration of therapeutic ^177^Lu, which is a more complex and irregular administration, that can take up to 30–40 min and entails several steps in which workers should approach radioactive source, and therefore are more prone to accidents, such as extravasation of radioactive liquid from the vial, patient movements during administration, vomiting, etc. This aspect not only introduces greater variability between the results of different sessions and workers, which is why the doses received at each position show a wide interquartile range (Fig. 6), but also leads to a more homogeneous exposure between the D and ND hand compared to the administration of a diagnostic radiopharmaceutical.

It was found that the wrist and ring CND dosemeters underestimated the maximum dose values. Nevertheless, this underestimation is expected, as they are located on the wrist and at the base of the ring finger, so it does not mean that these dosemeters perform poorly. In fact, the values recorded with the rings resulted similar to the values recorded with the TLDs located at the same position (e/E) in almost all the dosemeter sets. This is also reflected in the value of the CFs (Table 7) which is similar for the ring and positions e/E. In the case of the wrist detectors, the CFs show larger differences, as expected, except for the nurse’s D hand. Thus, as set out by other studies, for measuring extremity doses in NM the use of ring over wrist dosemeter is recommended [25, 47].

The CFs, or dose ratios, were calculated at the base of the middle (d/D) and ring (e/E) fingers, as these are the most common positions for placing a ring dosemeter. The calculated median CFs suggest that physicians should correct the middle and ring doses by at least a factor of 5 and 6 respectively, and nurses by a factor of 3 and 4 respectively, preferably located on the ND hand. To the best of our knowledge, and according to Kollaard et al. [26] only one publication has reported on dose ratios [24], obtaining a value of 1.6, which is very different from our results. However, this value was calculated by averaging the dose ratios over the three measured fingers (thumb, index and middle), which may underestimate the maximum CFs. In addition, the estimated values are similar to those generally recommended in the literature for the assessment of maximum finger doses in nuclear medicine [50].

It should be noted that, compared to the ORAMED project [25, 38, 51], the position of a TLD at the base of the index finger was not taken into account in this study, a factor that could have potentially yielded more detailed insights into the dose distribution over the hands. However, it is noteworthy that in everyday clinical practice, the ring dosemeter is not exclusively worn on the index finger. In fact, through an ongoing measurement campaign within the SINFONIA project in various European hospitals, the base of the middle finger was one of the observed positions, also consistent with our hospital's practice. Subsequently, during the same campaign a comparison of the dose at the base of the middle and ring finger to the dose at the base of the index finger of the non-dominant hand was performed. It was obtained that the ratio index/ring finger showed a mean of 1.2 [0.6–2.2] and the ratio index/middle a mean of 1.1 [0.6–1.9]. This implies that the mean difference between these two monitored positions and the base of the index finger is approximately 10–20%, falling within the range of 0.60–2.2. Therefore, although it could have provided additional insights on the dose distribution, the absence of TLD data from the base of the index finger is relatively inconsequential due to minor dose variations among the base of these three fingers, underscored by the fact that the index finger is not the exclusive position where the ring dosemeters are worn in routine clinical practice.

Regarding doses to the eyes in terms of Hp(3)/A, a median of 2.02 [1.84–2.20] µSv/GBq and 1.76 [1.00–2.53] µSv/GBq was found for physicians and nurses, respectively. No significant differences were found between left and right eyes, although given the isotropic nature of gamma emission, it is expected. No studies on the estimation of Hp(3), especially from ^177^Lu management, were found to compare our results, so further measurements are needed to validate them, as there may be large inter-worker variability. Nevertheless, these first measurements indicate that there is no large concern to reach the annual eye dose limits.

Finally, annual dose estimates were made in terms of the maximum number of sessions and patients expected to be treated within safe dose limits, wearing a 0.5 mm lead equivalent apron. Outliers were also included in these calculations, as they inevitably represent the potential hazards faced by the staff in real treatments, such as cross-contamination or unexpected irradiation. With outliers, the most restrictive value was found to be a limit of 59 sessions/year for P2 due to skin dose, followed by 100 sessions/year for P1 due to eye dose and to 197 for P1 due to Hp(10)/A recorded with OSLs. Assuming that a patient requires 4 sessions of 7.4 GBq each, administered 8 weeks apart in the same year, the annual limit of patients per worker according to these values would be 15, 25 and 49 due to Hp(0.07), Hp(3) and Hp(10), respectively. Without outliers and OSL Hp(10) measurements, the session (patient) limit per worker would increase up to 965 (241), limited by the skin dose. In our hospital 21 sessions were performed in 2022 (12 by P1 and 9 by P2), involving 7 patients, so even accounting with outliers, dose limits were not reached due to ^177^Lu treatments. Nevertheless, the number of annual patients in other hospital has been found to be larger in the literature, from 11 to 25 patients per worker [4, 24], so the risk of overexposure should be considered in these cases. Besides, in out hospital ^177^Lu-DOTATATE is received in individual patient doses, so only administration is monitored (from pre to post activity verification), while in other hospitals also preparation and dispensing could be performed in-house. On top of that, the real situation would be more complex as these estimations only refer to ^177^Lu-DOTATATE treatments and usually one worker would perform several procedures with multiple radionuclides. In addition, as treatments with ^177^Lu-PSMA-617 are expected to start in the near future, exposure to ^177^Lu will increase and must be taken into account. It is therefore particularly important to ensure that staff dosimetry is adequate to ensure that ICRP limits are not exceeded.

In addition, it is worth noting that the measured activity of each vial averaged 7121 ± 105 MBq, ranging from 6808 to 7289 MBq, which is less than the intended activity of 7400 MBq/cycle. This difference can be explained because all radiopharmaceuticals, including Lutathera vials, are distributed regionally from a central radiopharmacy to hospitals. In the studied hospital, Lutathera vials are shipped from the factory to the radiopharmacy one day before treatment, then transported to the hospital on the treatment day without any handling at the radiopharmacy. Although the process is carefully planned so that the vial reaches the hospital with an activity level of 7400 MBq at the time determined by the treating physician, unforeseen circumstances and external factors beyond the control of the staff, such as delays in delivery or patient arrival, can lead to small variations in the vial's activity level, causing a 4–5% variance from the target of 7400 MBq.

This study has thoroughly analysed the doses received by workers administering ^177^Lu-DOTATATE and has shown that its results are consistent with the limited existing literature. However, it presents some limitations. Firstly, there is no information on post-administration dose rates (4, 6 or 24 h after administration), which would be of great interest as staff may also approach the patient during this period. However, based on other studies, these doses are rather low [14, 23]. It should be noted that the inpatient basis was chosen for internal organisation and logistic reasons, but it would be possible to perform the administration on an outpatient basis, as suggested by other authors [14, 52], under the requirement that the patient returns within 24 h of administration for the scan and given that it complies with RP measures. The relationship between the usual ring and wrist dosemeters and TLDs was also investigated, but the results would have been more robust if they had been worn on either the D or ND hand, rather than both. However, this reflects the reality of staff dose monitoring, as workers often wear the detectors in different positions. On the other hand, the effect of the lead apron was studied by comparing dose rates and whole-body doses received with and without the apron using the real-time dosemeter, but a more complete analysis would have required double dosimetry (one dosemeter above and one below the apron), as has been done in other studies [49]. However, this approach was not possible as only one electronic dosemeter was available for the physician and one for the nurse, as well as only one OSL in each set used. The number of sessions performed without apron was also limited, so as mentioned before, a thorough hypothesis testing was not possible. In addition, the study covers data from 2 physicians and 4 nurses, but a larger sample size could provide more statistical power and add information on those dosemeters for which few results were obtained, such as OSLs. Finally, some values were reported as outliers and attributed to possible cross-contamination, but it is not possible to be sure that this is indeed the reason for these high values. Therefore, the effect of cross-contamination should be investigated in the future and compared with the values obtained in this study.

## Conclusion

The increasing production of therapeutic radiopharmaceuticals emphasises the need to control the doses received by nuclear medicine professionals involved in these practices. Particularly, attention must be paid to ^177^Lu due to its dual beta and gamma emission. The use of ^177^Lu-DOTATATE for the treatment of neuroendocrine tumours is increasing, as will be future treatments with ^177^Lu-based radiopharmaceuticals, such as ^177^Lu-PSMA-617 for prostate cancer. This study conducted a comprehensive analysis of the doses received by nuclear medicine staff involved in the administration of ^177^Lu-DOTATATE using multiple dose equivalents obtained with both passive and active dosimetry. Dose ratios (CFs) between the maximum doses to the hands and the dose received at the base of the fingers were established to ensure accurate assessment of hand doses with routine ring dosemeters. The results indicate that the procedure is safe for workers if good practices are followed, such as minimising unshielded exposure time near the vial or patient and employing a 0.5 mm lead apron. Nonetheless, workers should be monitored to ensure that the annual dose limits are not exceeded, which can happen if cross-contamination occurs.

### Supplementary Information

**Additional file 1**. The available supplementary material provides additional details on the nuclear medicine facilities and materials used for the administration of ^177^Lu-DOTATATE. Additional tables with complementary statistical information are also included.

## Data Availability

The datasets used and analysed during the current study are available from the corresponding author on reasonable request.

## List of abbreviations

^131^I: Iodine-131
^177^Hf: Hafnium-177
^177^Lu: Lutetium-177
^18^F: Fluor-18
^68^Ga: Gallium-68
^90^Y: Yttrium-90
A: Total measured activity
CF: Correction factor
CND: Spanish National Dosimetry Centre
D: Dominant hand
DOTATATE: DOTA-Tyr3-octreotate
DOTATOC: DOTA-Phe1-Tyr3-octreotide
EMA: European Medicines Agency
FDA: US Food and Drug Administration
GEP: Gastroenteropancreatic
IQR: Interquartile range
IR: Infusion rate
LDL: Lowest detection limit
LiF: Lithium fluoride
NaCl: Sodium chloride
ND: Non-dominant hand
NET: Neuroendocrine tumours
NM: Nuclear medicine
OSL: Optically stimulated luminescence
PED: Personal electronic dosemeters
PET: Positron emission tomography
PMMA: Polymethyl methacrylate
PRRT: Peptide receptor radionuclide therapy
PSMA: Prostate-specific membrane antigen
SCK CEN: Belgian Nuclear Research Centre
SD: Standard deviation
SSTR: Somatostatin receptor
TLD: Thermoluminescent dosemeters
