# Supplementary material for: Radiation exposure assessment of nuclear medicine staff administering [177Lu]Lu-DOTA-TATE with active and passive dosimetry
Source: EJNMMI Phys. 2023 Nov 14;10:70. doi: 10.1186/s40658-023-00592-1 (PMC10645926; doi:10.1186/s40658-023-00592-1)
Supplement: Supplementary file 1 — Additional file 1. The available supplementary material provides additional details on the nuclear medicine facilities and materials used for the administration of 177Lu-DOTATATE. Additional tables with complementary statistical information are also included. [file 40658_2023_592_MOESM1_ESM.pdf]

Radiation Exposure Assessment of Nuclear Medicine Staff Administering [^{177}Lu]Lu-DOTA-TATE with Active and Passive Dosimetry - *Supplementary material*

Mercedes Riveira-Martin[1,2*], Lara Struelens[3], José Muñoz Iglesias[4], Werner Schoonjans[3], Olga Tabuenca[4], José Manuel Nogueiras[5], Francisco Javier Salvador Gómez[6], Antonio López Medina[6,7]

[1]Genetic Oncology, Radiobiology and Radiointeraction Research Group, Galicia Sur Health Research Institute, Vigo, Spain

[2]Department of Radiology, Rehabilitation and Physiotherapy, Medicine School, Complutense University of Madrid, Madrid, Spain.

[3]Belgian Nuclear Research Centre (SCK CEN), Mol, Belgium.

[4]Meixoeiro Hospital, University Hospital of Vigo, Nuclear Medicine Department (SERGAS), Vigo, Spain.

[5]Meixoeiro Hospital, University Hospital of Vigo, Nuclear Medicine Department (GALARIA), Vigo, Spain.

[6]Meixoeiro Hospital, University Hospital of Vigo, Medical Physics and RP Department (GALARIA), Vigo, Spain.

[7]Department of functional Biology and Health Sciences, University of Vigo, Spain.

Corresponding author

Mercedes Riveira-Martin*

Galicia Sur Health Research Institute (IISGS)

Genetic Oncology, Radiobiology and Radiointeraction Research Group

Tel: +34 618 443 007

Email: mercedes.riveira@iisgaliciasur.es

Supplementary material:

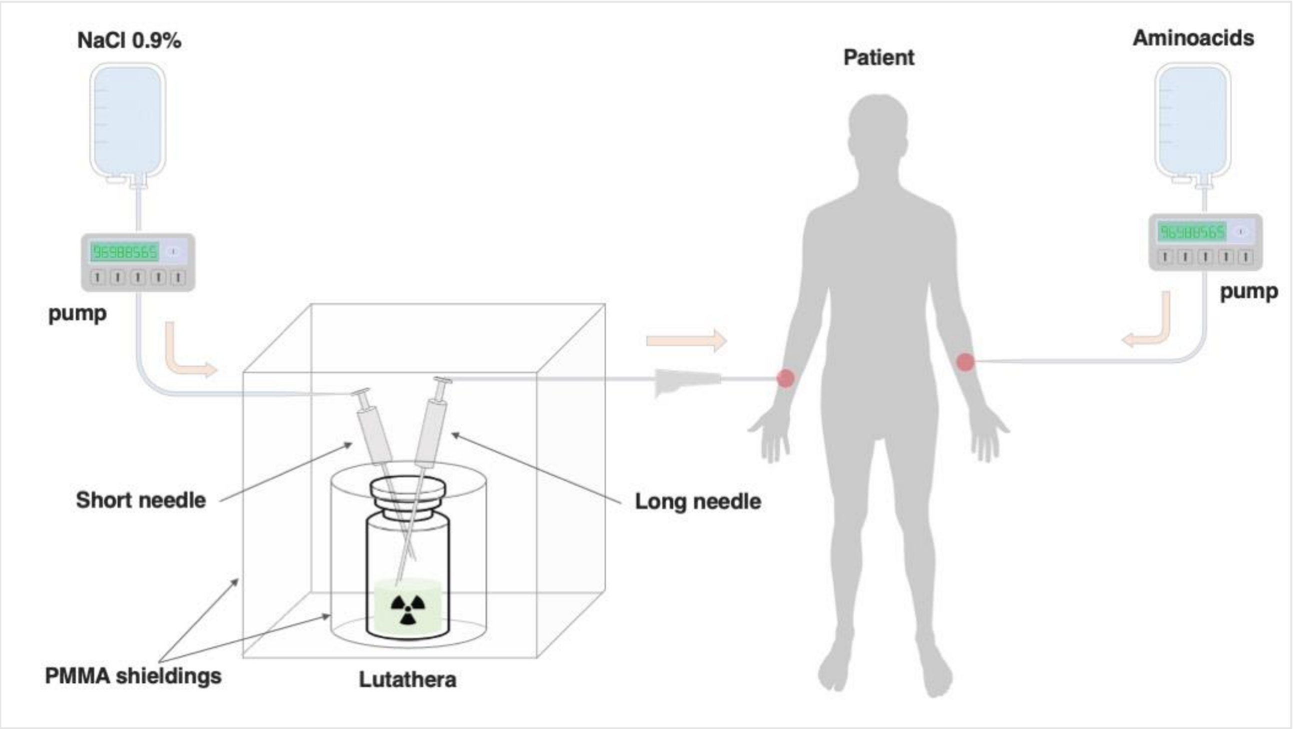

Fig. S1 Schematic representation of the gravity method used for infusion of Lutathera.

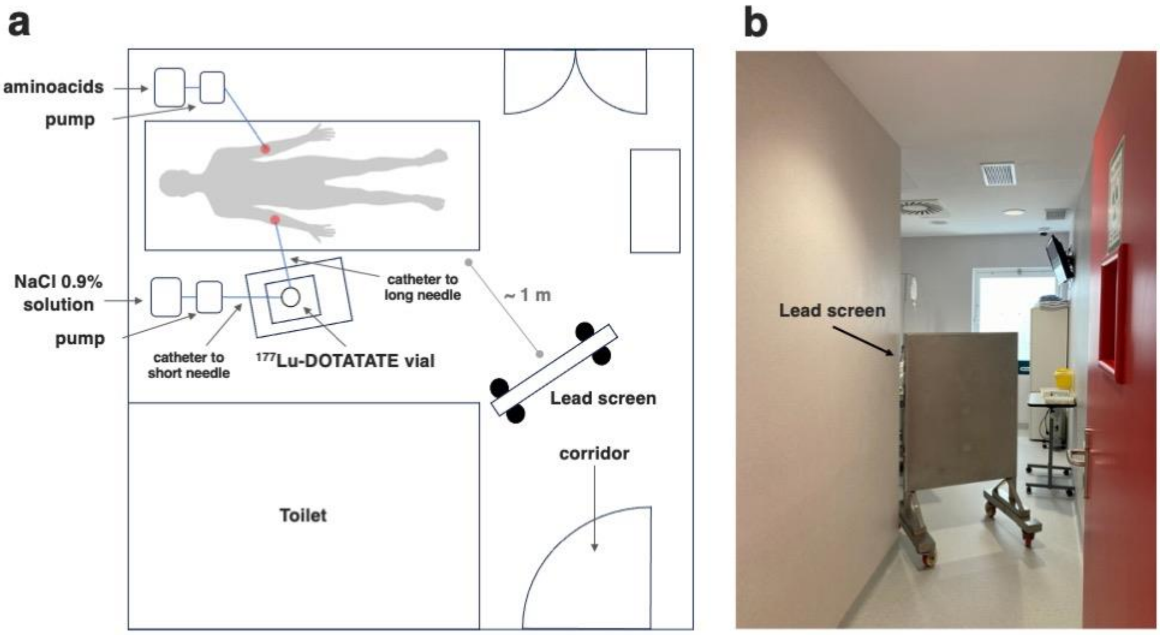

Fig. S2. Map of the administration room illustrating the most common positions of every item (a) and picture of the administration room showing the lead screen (b)

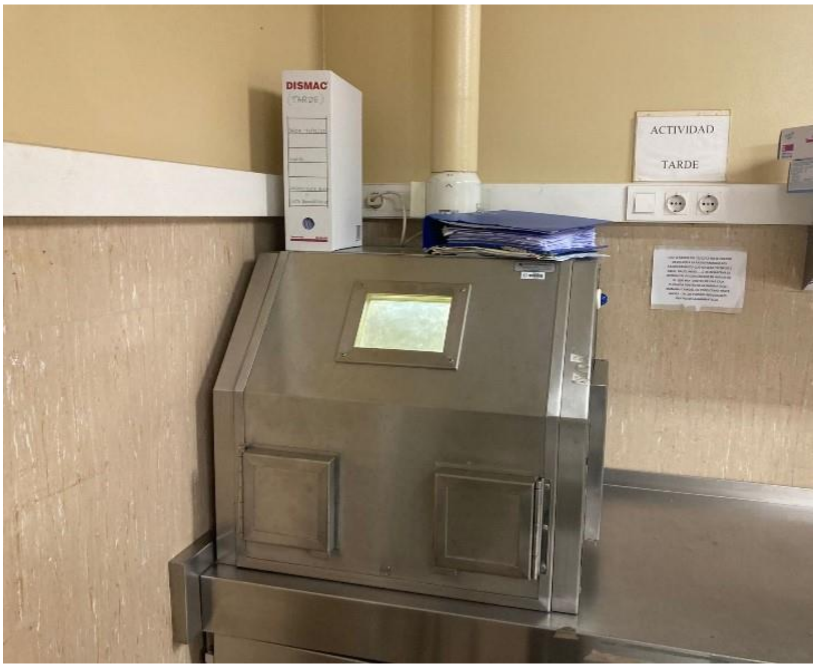

Fig. S3. Shielded cabinet in which the lutetium vial is left in its original lead shielding for 6 months until it has decayed.

Table S1 Data recorded with PEDs for P1 and N1 from the sessions performed with and without lead apron. The table shows the number of sessions monitored, the time of treatment (from the administration to the end of treatment), the maximum dose rate reached, and the cumulative dose normalized to the handled activity. N = 29 sessions were monitored with apron for both physician and nurse, whereas N = 2 and N = 3 sessions for the physician and nurse, respectively, without apron. Results are shown as median [range].

Staff	Apron	Time (min)	Dose rate peak (µSv/h)	Hp(10)/A (µSv/GBq)
**P1**	No	55 [49 - 61]	253.1 [222.4 - 283.8]	1.32
	Yes	48 [29 - 68]	74.6 [55.1 - 163.9]	0.56
**N1**	No	46 [40 - 52]	319.2 [186 - 433.8]	2.97
	Yes	46 [33 - 56]	140.6 [43.9 - 259]	0.97

Table S2 Mean values of residual activity, maximum dose rate, effective normalized dose Hp(10)/A (both measured with PEDs), and the time spent in the treatment, for each worker and divided by method. The *P-value* from the Mann-Whitney U-test is shown for each pair of measurements.

Variables / Staff	Mean ± SD		*P-value*
	Protocol 1	Protocol 2	
**Residual activity (MBq)**			
P1	49.4 ± 7.1	34 ± 6.8	< 0.01*
P2	68.5 ± 21.6	56.5 ± 7.5	0.3
**Max. Dose rate (µSv/h)**			
P1	82.9 ± 22.4	98.8 ± 41.3	0.7
P2	93.3 ± 14.6	108.8 ± 43.6	0.5
N1	117.5 ± 97.5	173.6 ± 42	0.3
N3	91.1 ± 38.4	123.6 ± 46	0.2
**Hp(10)/A (µSv/GBq)**			
P1	0.6 ± 0.4	0.5 ± 0.4	0.7
P2	0.7 ± 0.2	0.6 ± 0.3	0.9
N1	0.8 ± 0.5	1.1 ± 0.6	0.7
N3	0.6 ± 0.4	0.4 ± 0.1	0.1
**Time (min)**			
P1	54.6 ± 7.7	36.6 ± 8.1	< 0.01*
P2	52.2 ± 8.3	36.6 ± 4.6	0.01*
N1	50.5 ± 5.3	39.0 ± 7.6	0.04*
N3	51.2 ± 6.3	34.3 ± 6.2	< 0.01*

*Statistically significant

Table S3 *P-values* of the Mann-Whitney U test to examine whether there are statistically significant differences between D and ND hand doses (Hp(0.07)/A) for each location for both physicians and nurses.

Group of workers	TLD location (ND vs. D)	*P-value*
Physicians	a/A	0.43
	b/B	0.06
	c/C	0.31
	d/D	0.82
	e/E	0.31
Nurses	a/A	0.69
	b/B	0.89
	c/C	0.69
	d/D	0.49
	e/E	0.23

Table S4 *P-values* of the Mann-Whitney U test examine whether Hp(0.07)/A doses in each position (D and ND hands) are significantly different between physicians and nurses.

Hand	TLD position (Physician vs. Nurse)	*P-value*
ND	a	0.01*
	b	0.04*
	c	0.02*
	d	0.01*
	e	0.02*
D	A	0.03*
	B	0.01*
	C	0.01*
	D	0.09
	E	0.48

*Statistically significant

Table S5 Measured normalised Hp(0.07) values for each position, set and worker along with the mean value over all sets for each worker. Median Hp(0.07)/A for each position is shown, as well as the median over the maximum values by hand for each group of workers.

Staff	set	Hp(0.07)/A (μSv/GBq)											
		TLD (Gloves)										CND	
		D					ND						
		**A**	**B**	**C**	**D**	**E**	**a**	**b**	**c**	**d**	**e**	**Ring**	**Wrist**
P1	#1	9.6	18.7	13.1	0.0	< LDL	41.4	30.7	23.3	13.0	28.8	< LDL	6.9
	#2	17.4	20.4	21.9	11.4	12.2	30.1	65.7	21.1	12.5	15.0	17.7	10.6
	#3	< LDL	20.2	13.9	9.1	7.8	34.2	47.0	14.4	12.0	9.9	14.0	7.0
	#4	25.3	20.9	17.6	13.7	8.4	40.7	53.5	19.4	11.2	8.8	14.0	4.0
	Mean of all sets	17.4	**20.1**	16.6	8.6	9.5	36.6	**49.2**	19.6	12.2	15.6	**15.2**	**7.1**
P2	#1	70.3	25.5	20.2	14.9	12.0	31.8	28.0	33.8	13.2	11.2	8.4	8.4
	#2	152.6*	1145.3*	155.9*	27.5*	61.9*	901.1*	163*	196.2*	38.2*	93.3*	108.8*	39.9*
	Mean of all sets	**70.3**	25.5	20.2	14.9	12.0	31.8	28.0	**33.8**	13.2	11.2	**8.4**	**8.4**
N1	#1	< LDL	< LDL	< LDL	< LDL	< LDL	< LDL	< LDL	< LDL	< LDL	< LDL	< LDL	< LDL
	#2	6.8	13.9	6.1	6.4	4.4	11.8	33.1	8.4	8.1	5.8	10.6	3.5
	Mean of all sets	6.8	**13.9**	6.1	6.4	4.4	11.8	**33.1**	8.4	8.1	5.8	**10.6**	**3.5**
N2	#1	14.8	10.9	11.7	< LDL	9.6	12.8	13.1	15.4	10.4	163.8*	< LDL	14.2
	Mean of all sets	**14.8**	10.9	11.7	-	9.6	12.8	13.1	**15.4**	10.4	-	-	**14.2**
N3	#1	8.6	8.1	5.5	6.6	11.1	8.5	6.9	7.2	4.6	3.0	7.8	4.7
	#2	7.8	10.9	6.8	4.1	3.6	8.5	7.9	4.6	3.5	3.4	61.4*	2.4
	Mean of all sets	8.2	9.5	6.2	5.4	7.4	8.5	7.4	5.9	4.1	3.2	**7.8**	**3.5**

Median Hp(0.07)/A by position (μSv/GBq)

	**A**	**B**	**C**	**D**	**E**	**a**	**b**	**c**	**d**	**e**	Ring	Wrist
**Physicians**	21.3	20.4	17.6	12.6	10.2	34.2	47.0	21.1	12.5	11.2	14.0	7.1
**Nurses**	8.2	10.9	6.5	6.4	7.0	10.1	10.5	7.8	6.3	3.4	12.3	4.1

Median of maximum values Hp(0.07)/A by hand (μSv/GBq)

	**D**	**ND**	**Ring**	**Wrist**
**Physicians**	45.2 [20 – 70.3]	41.5 [33.8 - 49.2]	11.8 [8.4 - 15.2]	7.8 [7.1 - 8.4]
**Nurses**	13.9 [9.5 - 14.8]	15.4 [8.5 - 33.1]	9.2 [7.8 - 10.6]	3.5 [3.5 - 3.5]

*Values considered outliers

The LDL for OSL dosemeters is 50 μSv and for ring and wrist dosemeters is 0.1 mSv.

Bold values represent the highest value assigned to each worker, which are used to calculate the median of the maximum Hp(0.07)/A values.
